# Sleep, Depressive Symptoms, and Quality of Life Among Women With Newly Diagnosed Breast Cancer: Baseline Results From the AMBER Cohort Study

**DOI:** 10.1002/cam4.71586

**Published:** 2026-02-12

**Authors:** Lin Yang, Temoor Tayyab, Renée L. Kokts‐Porietis, Qinggang Wang, Jessica McNeil, Charles E. Matthews, Leanne Dickau, Jeff K. Vallance, Margaret L. McNeely, S. Nicole Culos‐Reed, Karen A. Kopciuk, Kerry S. Courneya, Christine M. Friedenreich

**Affiliations:** ^1^ Department of Cancer Epidemiology and Prevention Research Cancer Care Alberta Calgary Alberta Canada; ^2^ Departments of Oncology and Community Health Sciences, Cumming School of Medicine University of Calgary Calgary Alberta Canada; ^3^ Department of Kinesiology, School of Health and Human Sciences University of North Carolina at Greensboro Greensboro North Carolina USA; ^4^ Division of Cancer Epidemiology and Genetics US National Cancer Institute Rockville Maryland USA; ^5^ Faculty of Health Disciplines Athabasca University Athabasca Alberta Canada; ^6^ Faculty of Rehabilitation Medicine University of Alberta Edmonton Alberta Canada; ^7^ Faculty of Kinesiology University of Calgary Calgary Alberta Canada; ^8^ Department of Mathematics and Statistics University of Calgary Calgary Alberta Canada; ^9^ Faculty of Kinesiology, Sport, and Recreation, College of Health Sciences University of Alberta Edmonton Alberta Canada

**Keywords:** breast cancer, depressive symptoms, intervention design, quality of life, sleep

## Abstract

**Background:**

Sleep problems are common among breast cancer survivors and are associated with poor quality of life. However, few studies have considered whether depressive symptoms confound or modify this association. This study evaluates whether depressive symptoms attenuate the association between sleep and quality of life in newly diagnosed breast cancer survivors.

**Methods:**

Women with newly diagnosed early‐stage breast cancer were recruited between 2012 and 2019 in Alberta, Canada, and completed the Pittsburg Sleep Quality Index (PSQI) to characterize global sleep quality and subscales. Quality of life was measured using the Short Form Survey (SF‐36 version‐2) to assess physical and mental well‐being. Depressive symptoms were measured using the Patient Health Questionnaire (PHQ‐9). Multivariable linear regressions were used to estimate the association of sleep characteristics with physical and mental well‐being. Depressive symptoms were evaluated as a potential confounder and effect modifier.

**Results:**

Among 1454 breast cancer survivors with available data, 43% reported poor global sleep quality and 10.5% reported clinically meaningful depressive symptoms. Poor sleep quality was associated with lower physical (*β* = −3.0, 95% CI: −3.8 to −2.3) and mental well‐being (*β* = −5.7, 95% CI: −6.7 to −4.7). These associations were attenuated to (*β* = −2.3, 95% CI: −3.1 to 1.5) and (*β* = −1.2, 95% CI: −2.1 to −0.3) after further adjusting for depressive symptoms. Stratified analyses showed slightly stronger associations in women with non‐minimal depressive symptoms, though this association was not clinically meaningful.

**Conclusion:**

The association between sleep and quality of life was substantially attenuated after accounting for depressive symptoms. Longitudinal data are needed to clarify whether depressive symptoms confound, mediate, or modify this relationship to better guide targeted interventions in breast cancer survivorship.

## 1 Introduction

Breast cancer is the most commonly diagnosed cancer among women worldwide, with an estimated 2.3 million new cases reported in 2022 [1]. Advances in early detection and clinical treatment have led to 5‐year survival rates exceeding 90% in high income countries [2]. Consequently, the breast cancer survivor population is rapidly growing. Many patients experience persistent health challenges that affect their quality of life (QoL).

Sleep problems are among the most common and persistent concerns in breast cancer survivorship [3]. Compared to the general population, sleep problems are nearly twice as prevalent among cancer survivors, reported by 30%–75% of those with newly diagnosed cancers [4]. These issues can persist in up to 40% of breast cancer survivors 5 years after diagnosis [5]. Previous studies have demonstrated a strong association between poor sleep quality and lower QoL among breast cancer survivors [6, 7, 8, 9, 10, 11, 12]. However, despite a well‐recognized bi‐directional relationship between sleep and depression in both the general population and among breast cancer survivors [13, 14, 15], few studies have accounted for depressive symptoms when examining associations between sleep and QoL in breast cancer survivors. As a result, current interventions typically target sleep problems and depressive symptoms in isolation [16, 17], overlooking their complex interplay. Without clarifying whether depressive symptoms function as a confounder, effect modifier, or mediator in the relationship between sleep and QoL, intervention strategies risk being poorly targeted.

In addition, sleep is a multidimensional construct that includes sleep duration, sleep timing, and sleep quality, each of which may uniquely influence physical and mental well‐being [18]. Previous research has often focused on insomnia as a single dimension of sleep [19, 20] or a global sleep quality score [12, 21], but few examined individual dimensions of sleep in relation to QoL.

To address these knowledge gaps, the present study investigates associations between global sleep quality and QoL, accounting for depressive symptoms as a potential confounder and effect modifier. In addition, we explore multiple dimensions of sleep quality (latency, efficiency, disturbance, medication use, and daytime dysfunction) in relation to physical and mental well‐being among women newly diagnosed with breast cancer. We hypothesize that poorer sleep quality will be associated with lower QoL, and that adjustment for depressive symptoms will attenuate but not fully eliminate this association. We further hypothesize that associations with QoL will differ across dimensions of sleep quality. Understanding these relationships is essential to inform intervention design and to determine whether sleep, depressive symptoms, or their joint targeting is most likely to improve QoL among breast cancer survivors.

## 2 Methods

### 2.1 Study Design and Participants

The Alberta Moving Beyond Breast Cancer (AMBER) study is a prospective population‐based cohort of 1528 women newly diagnosed with incident, stage I–IIIc breast cancer between July 2012 and July 2019 in Edmonton or Calgary, Alberta, Canada, including surrounding areas. Full cohort and eligibility details for AMBER have been previously described [1]. Briefly, eligible participants were women aged 18 to 80 years with histologically confirmed primary breast cancer who were not pregnant at the time of recruitment, were able to complete study questionnaires in English, and met safety criteria for participation as assessed using the revised Physical Activity Readiness Questionnaire for Everyone (PAR‐Q+). In Calgary, potential participants were identified through the Alberta Cancer Research Biobank, which approached all newly diagnosed breast cancer patients at the time of diagnosis. Women who consented to be contracted for research were subsequently contacted for the AMBER cohort study once their clinical and pathology information became available to confirm eligibility. In Edmonton, eligible participants were identified through the Cross Cancer Institute's New Patient Breast Cancer clinics and were introduced to the study by their treating oncologist during their first clinic visit. Women who expressed interest were further screened for eligibility and contacted by telephone to confirm participation. Ethics approval was obtained through the Health Research Ethics Board of Alberta: Cancer Committee (HREBA.CC‐17‐0576). All participants provided informed, written consent.

### 2.2 Data Collection

All variables in the study were derived from baseline assessments of participants that were completed within 90 days of surgery [22]. Participants' demographic information and medical histories were captured with a self‐reported questionnaire including details on marital status, self‐reported ethnicity, education attainment, annual family income, smoking status, and comorbidities. Comorbidity scores were determined using the Charlson Comorbidity Index [23]. Past year alcohol consumption and total dietary intake were assessed with the Canadian Diet History Questionnaire‐II [24]. Anthropometric measurements were directly measured by trained staff, and body composition was assessed with dual x‐ray absorptiometry. Physical activity was assessed through the self‐administered Past Year Total Physical Activity Questionnaire [25] and through direct measurement with an ActiGraph GT3‐X accelerometer for 1 week. In addition, all pathologic and clinical aspects of each participant's cancer, treatment and follow‐up care were obtained through medical chart abstractions done by a trained staff member.

### 2.3 Sleep Characteristics

The Pittsburgh Sleep Quality Index (PSQI) questionnaire captures multiple dimensions of sleep, including a global sleep quality score based on the past 30 days [26]. The PSQI global score is a sum of subscales across seven dimensions: subjective sleep quality, sleep latency, sleep duration, habitual sleep efficiency, sleep disturbances, use of sleep medication, and daytime dysfunction. Each individual subscale ranges from 0 to 3, with three indicating the greatest severity. The global score ranges from 0 to 21, with higher scores indicating poorer sleep quality.

The PSQI global score was dichotomized based on a cutoff score of 8 to characterize good sleepers (PSQI global score ≤ 8) and poor sleepers (PSQI global score > 8) [27]. While a cutoff score of 5 is commonly used in general populations, previous research suggests that a threshold of 8 serves as a more accurate indicator for identifying individuals with sleep problems within clinical populations, including those with breast cancer [27]. The PSQI subscales were grouped according to the instructions for this questionnaire [27], apart from sleep duration which was classified to align with recent literature (7–9 h/day, < 7 h/day, ≥ 9 h/day) [28]. Subscales without PSQI‐characterized category labels (e.g., sleep latency, sleep disturbance, daytime dysfunction) were classified into minimal, mild, moderate, and severe. Besides PSQI‐defined subscales, sleep onset timing (< 10 p.m., 10–11 p.m., 11–12 p.m., ≥ 12 p.m.) [29, 30] was also included to determine if this sleep characteristic is associated with QoL.

### 2.4 Depressive Symptoms

Depressive symptoms were measured using the Patient Health Questionnaire‐9 (PHQ‐9), a validated 9‐item depression screener asking about the frequency of symptoms of depression over the past 2 weeks [18]. Each item was scored on a 0–3 scale. The total score of the PHQ‐9 ranged from 0 to 27 and was categorized as “none or minimal” (0–4), “mild” [5, 6, 7, 8, 9], “moderate” [10, 11, 12, 13, 14], “moderately severe” [15, 16, 17, 18, 19], and “severe” [20, 21, 22, 23, 24, 25, 26, 27]. PHQ‐9 score of 10 has been widely used for detecting major depressive disorder, and a score under 5 represents remission [31]. For current analyses, participants who scored 5 or higher were combined into one group as having at least mild depressive symptoms.

### 2.5 Quality of Life

Health‐related QoL was measured with the 36‐item Short Form Survey (SF‐36) questionnaire at baseline [32]. It yields a comprehensive score for an individual's physical and mental health by providing a Physical Component Score (PCS) and Mental Component Score (MCS). SF‐36 is a function of eight health domains. Physical functioning, bodily pain, and role limitations are physical problems that mainly shape the PCS, while mental health and role limitations attributable to emotional problems primarily shape MCS. The remaining three domains—vitality, social functioning, and general health perceptions—contribute significantly to both physical and mental components [33]. PCS and MCS both range from 0 to 100. Lower scores indicate worse health‐related QoL, with 3–5 points difference being accepted as minimal clinically meaningful differences [34].

### 2.6 Statistical Analysis

Descriptive statistics compared sample characteristics overall and by global sleep quality (good vs. poor sleepers). Age‐adjusted and multivariable linear regressions were used to estimate *β* coefficients and 95% confidence intervals (95% CI) for the associations of sleep characteristics (global sleep quality and PSQI subscales) with PCS and MCS. Covariates were selected a priori based on previous literature (marital status, ethnicity, education level, alcohol consumption, smoking status, total caloric intake, disease stage, tumor stage, lean mass/fat mass ratio, moderate to vigorous physical activity, comorbidity score) [6, 8, 35] or specific to this study (e.g., study location). *P* trend was assessed with the continuous version for most of the PSQI variables in linear models.

With respect to depressive symptoms, we evaluated their potential role as a confounder by including them as a covariate in the multivariable regression models. To assess potential effect modification, we tested an interaction term between depressive symptoms and global sleep quality. Given a significant interaction with the MCS, we conducted stratified analyses comparing participants with no or minimal depressive symptoms to those with at least mild depressive symptoms. Additionally, sensitivity analyses were conducted to explore the associations of sleep with physical and mental well‐being in population subgroups defined by their treatment status: active treatment (during radiation and/or chemotherapy) and not on treatment (completed neo‐chemotherapy or had not started treatment) at the time of baseline measurements.

Participants with missing values on either the exposure or outcome of interest were excluded from the analyses (*n* = 73). For covariates with < 5% missingness, multivariate imputations were conducted via chained equations, incorporating all correlated covariates into regression models to prevent sample size reduction [36]. Analyses were performed using R (version 4.4.1, R Foundation for Statistical Computing, Vienna, Austria). Statistical significance level (*α*) of 0.05 was used in these analyses. Analyses were not adjusted for multiple testing and should be interpreted as exploratory in nature.

## 3 Results

### 3.1 Baseline Participant Characteristics

Of the 1528 participants in the AMBER cohort, 1454 participants had complete data on exposures and outcomes and were included in this analysis. The mean age of participants was 55.5 years, most participants were white (87.3%), married or common‐law (74.9%), had never smoked (57.1%), and had above a high school level of education and a household income above $50,000 (Table 1). Participants primarily had stage I (45.0%) or stage II (46.3%) breast cancer and were treated with lumpectomy (56.3%) or mastectomy (36.1%). Detailed information on adjuvant therapy, reproductive characteristics and PSQI subscale scores are reported in Tables S1 and S2. Characteristics of the participants not included in the analysis (*n* = 73) were not meaningfully different from those included (data not shown). Overall, 43% reported poor global sleep quality and 10.5% reported clinically meaningful depressive symptoms.

**TABLE 1:** Demographic, lifestyle and clinical characteristics and sleep quality of the AMBER cohort at baseline, 2012–2019 (*N* = 1454).^a^

	Overall (*N* = 1454)	Good sleeper (*N* = 828)	Poor sleeper (*N* = 626)
Location			
Calgary	829 (57.0%)	468 (56.5%)	361 (57.7%)
Edmonton	625 (43.0%)	360 (43.5%)	265 (42.3%)
Age			
Mean (SD)	55.5 (10.7)	55.7 (11.0)	55.3 (10.3)
Marital status			
Divorced, separated, widowed	264 (18.2%)	147 (17.8%)	117 (18.7%)
Married, common‐law	1089 (74.9%)	633 (76.4%)	456 (72.8%)
Single or never married	101 (6.9%)	48 (5.8%)	53 (8.5%)
Highest level of education			
High school or below	328 (22.6%)	174 (21.0%)	154 (24.6%)
College	463 (31.8%)	247 (29.8%)	216 (34.5%)
University	381 (26.2%)	243 (29.3%)	138 (22.0%)
Graduate school	282 (19.4%)	164 (19.8%)	118 (18.8%)
Income			
< $50,000	234 (16.1%)	117 (14.1%)	117 (18.7%)
$50,000–$99,999	464 (31.9%)	273 (33.0%)	191 (30.5%)
$100,000–$149,999	338 (23.2%)	186 (22.5%)	152 (24.3%)
> $150,000	418 (28.7%)	252 (30.4%)	166 (26.5%)
Ethnicity			
Non‐white	184 (12.7%)	106 (12.8%)	78 (12.5%)
White	1270 (87.3%)	722 (87.2%)	548 (87.5%)
Daily alcohol intake (g)			
Mean (SD)	7.15 (16.3)	6.63 (11.2)	7.83 (21.2)
Missing	11 (0.8%)	6 (0.7%)	5 (0.8%)
Daily caloric intake (kcal/day)			
Mean (SD)	1720 (748)	1670 (709)	1780 (793)
Missing	11 (0.8%)	6 (0.7%)	5 (0.8%)
Lean/fat mass ratio			
Mean (SD)	1.32 (0.431)	1.36 (0.460)	1.26 (0.383)
Missing	19 (1.3%)	10 (1.2%)	9 (1.4%)
Smoking status			
Current	122 (8.4%)	57 (6.9%)	65 (10.4%)
Former	502 (34.5%)	273 (33.0%)	229 (36.6%)
Never	830 (57.1%)	498 (60.1%)	332 (53.0%)
Moderate to intense physical activity (min/day)			
Mean (SD)	1.03 (0.575)	1.06 (0.584)	0.994 (0.562)
Missing	57 (3.9%)	36 (4.3%)	21 (3.4%)
Surgery status			
Lumpectomy	818 (56.3%)	477 (57.6%)	341 (54.5%)
Mastectomy	525 (36.1%)	287 (34.7%)	238 (38.0%)
Neoadjuvant	110 (7.6%)	63 (7.6%)	47 (7.5%)
Missing	1 (0.1%)	1 (0.1%)	0 (0%)
AJCC stage			
I	655 (45.0%)	370 (44.7%)	285 (45.5%)
II	673 (46.3%)	380 (45.9%)	293 (46.8%)
III	126 (8.7%)	78 (9.4%)	48 (7.7%)
Tumor grade			
1	185 (12.7%)	101 (12.2%)	84 (13.4%)
2	622 (42.8%)	371 (44.8%)	251 (40.1%)
3	646 (44.4%)	355 (42.9%)	291 (46.5%)
Missing	1 (0.1%)	1 (0.1%)	0 (0%)
Comorbidity score			
Mean (SD)	0.921 (1.04)	0.821 (0.923)	1.05 (1.17)

^a^ Good and poor sleepers were defined by global PSQI scores (obtained through the Pittsburgh Quality Index questionnaire), with values of 8 or less corresponding to good sleepers and values > 8 corresponding to poor sleepers.

### 3.2 Associations Between Sleep Characteristics and PCS/MCS

Tables 2 and 3 present age‐adjusted and multivariable adjusted models on the associations of sleep characteristics with PCS and MC, respectively.

**TABLE 2:** Associations of sleep characteristics with SF‐36 measured quality of life, Physical Component Score (PCS) in the AMBER cohort study (*N* = 1454).

	Mean PCS (SD)	Beta‐coefficient (95% CI)		
		Age‐adjusted^a^	MV model 1^a^, ^b^	MV model 2^a^, ^b^, ^c^
Sleep profile^d^				
Good sleepers	50.91 (7.11)	0 [reference]	0 [reference]	0 [reference]
Poor sleepers	47.00 (7.46)	−3.91 (−4.66, −3.15)	−3.09 (−3.83, −2.34)	−2.29 (−3.09, −1.49)
*p*‐trend		< 0.01	< 0.01	< 0.01
Sleep quality				
Very good	51.02 (7.62)	0 [reference]	0 [reference]	0 [reference]
Fairly good	49.89 (7.12)	−1.14 (−2.22, −0.06)	−1.09 (−2.14, −0.04)	−0.83 (−1.87, 0.21)
Fairly bad	47.36 (7.68)	−3.68 (−4.90, −2.46)	−2.86 (−4.06, −1.66)	−1.76 (−3.01, −0.52)
Very bad	45.59 (7.55)	−5.44 (−7.40, −3.49)	−4.55 (−6.43, −2.68)	−2.83 (−4.80, −0.88)
*p*‐trend		< 0.01	< 0.01	< 0.01
Sleep duration				
6–9 h/day	49.99 (7.19)	0 [reference]	0 [reference]	0 [reference]
≤ 6 h/day	47.99 (7.68)	−2.00 (−2.86, −1.14)	−1.43 (−2.27, −0.60)	−0.66 (−1.51, 0.18)
≥ 9 h/day	48.63 (8.21)	−1.35 (−2.61, −0.09)	−1.00 (−2.21, 0.20)	−0.72 (−1.90, 0.47)
Sleep timing				
< 10 p.m.	49.43 (7.39)	0 [reference]	0 [reference]	0 [reference]
10 to < 11 p.m.	49.51 (7.33)	0.08 (−0.97, 1.13)	−0.21 (−1.22, 0.79)	−0.25 (−1.24, 0.73)
11–< 12 p.m.	49.25 (7.56)	−0.19 (−1.29, 0.91)	0.19 (−0.87, 1.25)	0.25 (−0.79, 1.28)
≥ 12 p.m.	47.63 (8.17)	−1.82 (−3.33, −0.32)	−0.48 (−1.96, 1.00)	−0.43 (−1.88, 1.03)
*p*‐trend		< 0.01	0.42	0.47
Sleep latency				
None	50.44 (7.00)	0 [reference]	0 [reference]	0 [reference]
Mild	49.68 (7.58)	−0.75 (−1.76, 0.26)	−0.52 (−1.50, 0.45)	−0.19 (−1.15, 0.78)
Moderate	48.90 (7.53)	−1.54 (−2.69, −0.38)	−1.33 (−2.44, −0.22)	−0.82 (−1.93, 0.28)
Severe	47.11 (7.54)	−3.32 (−4.53, −2.11)	−2.18 (−3.35, −1.00)	−0.94 (−2.15, 0.28)
*p*‐trend		< 0.01	< 0.01	0.06
Sleep efficiency				
≥ 85%	50.40 (7.34)	0 [reference]	0 [reference]	0 [reference]
75%–84%	49.18 (7.57)	−1.23 (−2.16, −0.29)	−0.93 (−1.84, −0.02)	−0.76 (−1.65, 0.14)
65%–74%	48.02 (7.52)	−2.39 (−3.51, −1.26)	−1.71 (−2.79, −0.62)	−1.12 (−2.20, −0.04)
January	46.90 (7.20)	−3.50 (−4.67, −2.32)	−2.56 (−3.71, −1.41)	−1.68 (−2.84, −0.52)
*p*‐trend		< 0.01	< 0.01	< 0.01
Sleep disturbance				
Mild	50.87 (7.02)	0 [reference]	0 [reference]	0 [reference]
Severe	47.31 (7.61)	−3.57 (−4.32, −2.81)	−2.70 (−3.46, −1.95)	−2.03 (−2.81, −1.25)
*p*‐trend		< 0.01	< 0.01	< 0.01
Sleep medication				
Not during the past month	49.97 (7.47)	0 [reference]	0 [reference]	0 [reference]
Less than once a week	48.95 (6.74)	−1.02 (−2.30, 0.25)	−0.76 (−1.99, 0.47)	−0.62 (−1.82, 0.59)
Once or twice a week	48.15 (7.54)	−1.84 (−3.21, −0.46)	−1.17 (−2.51, 0.17)	−0.60 (−1.92, 0.73)
Three or more times a week	47.03 (7.66)	−2.95 (−4.01, −1.88)	−2.14 (−3.16, −1.10)	−1.48 (−2.51, −0.45)
*p*‐trend		< 0.01	< 0.01	< 0.01
Day time dysfunction				
None	52.19 (6.47)	0 [reference]	0 [reference]	0 [reference]
Mild	48.56 (7.48)	−3.65 (−4.48, −2.82)	−2.99 (−3.80, −2.18)	−2.43 (−3.28, −1.58)
Moderate	45.38 (7.27)	−6.88 (−8.27, −5.49)	−5.40 (−6.78, −4.02)	−4.11 (−5.66, −2.57)
Severe	41.41 (6.51)	−10.82 (−13.83, −7.81)	−9.55 (−12.58, −6.51)	−8.28 (−11.41, −5.14)
*p*‐trend		< 0.01	< 0.01	< 0.01

Abbreviations: CI, confidence intervals; MV, multivariable model.

^a^ Adjusted for age (years).

^b^ Multivariable (MV) model additionally adjusted study location (Edmonton, Calgary), marital status (married or common‐law, widowed/separated/divorced, single/never married), ethnicity (White, non‐white), education attainment (high school or below, college, university, graduate school), annual family income (< $50,000, $50,000–$100,000, $100,000–$150,000, $150,000), lean/fat mass ratio (kg/m^2^), total caloric intake (kcal/day), moderate to vigorous intensity physical activity (min/day), alcohol consumed (g/day), smoking (never smoker, past smoker, current smoker), disease stage (I, II, III), tumor grade (1, 2, 3), surgery status (pre‐surgery [neoadjuvant therapy], lumpectomy, mastectomy), and comorbidity score (0–8) obtained from the Charlson Comorbidity Index.

^c^ Additionally adjusted for depressive symptoms (0–21) obtained from the Patient Health Questionnaire (PHQ‐9).

^d^ Good and poor sleepers were defined by global PSQI scores (obtained through the Pittsburgh Quality Index questionnaire), with values of 8 or less corresponding to good sleepers, and values > 8 corresponding to poor sleepers.

**TABLE 3:** Associations of sleep characteristics with SF‐36 measured quality of life, Mental Component Score (MCS).

	Mean MCS (SD)	Beta‐coefficient (95% CI)		
		Age‐adjusted^a^	MV model 1^a^, ^b^	MV model 2^a^, ^b^, ^c^
Sleep profile^d^				
Good sleepers	50.55 (8.95)	0 [reference]	0 [reference]	0 [reference]
Poor sleepers	44.29 (10.23)	−6.16 (−7.12, −5.20)	−5.68 (−6.69, −4.67)	−1.16 (−2.06, −0.27)
*p*‐trend		< 0.01	< 0.01	< 0.01
Sleep quality				
Very good	52.68 (8.57)	0 [reference]	0 [reference]	0 [reference]
Fairly good	49.05 (9.09)	−3.42 (−4.77, −2.07)	−2.98 (−4.38, −1.57)	−1.83 (−3.00, −0.67)
Fairly bad	43.62 (10.26)	−8.58 (−10.11, −7.06)	−7.67 (−9.28, −6.06)	−2.44 (−3.83, −1.06)
Very bad	40.24 (11.01)	−11.97 (−14.41, −9.54)	−11.37 (−13.89, −8.85)	−2.28 (−4.47, −0.09)
*p*‐trend		< 0.01	< 0.01	< 0.01
Sleep duration				
6–9 h/day	49.34 (9.23)	0 [reference]	0 [reference]	0 [reference]
≤ 6 h/day	45.25 (10.68)	−3.90 (−5.00, −2.79)	−3.75 (−4.89, −2.60)	−0.37 (−1.31, 0.58)
≥ 9 h/day	47.07 (10.61)	−1.90 (−3.52, −0.28)	−2.30 (−3.95, −0.64)	−1.02 (−2.35, 0.30)
Sleep timing				
< 10 p.m.	47.25 (10.18)	0 [reference]	0 [reference]	0 [reference]
10–11 p.m.	48.03 (9.95)	0.79 (−0.58, 2.16)	0.35 (−1.05, 1.75)	−0.01 (−1.11, 1.08)
11–12 p.m.	48.44 (9.58)	0.95 (−0.48, 2.38)	0.71 (−0.77, 2.18)	0.79 (−0.37, 1.95)
≥ 12 p.m.	46.58 (11.15)	−1.08 (−3.03, 0.88)	0.00 (−2.06, 2.06)	0.07 (−1.55, 1.70)
*p*‐trend		0.03	0.50	0.59
Sleep latency				
None	51.17 (9.08)	0 [reference]	0 [reference]	0 [reference]
Mild	48.48 (9.54)	−2.53 (−3.81, −1.25)	−2.75 (−4.07, −1.43)	−1.53 (−2.60, −0.45)
Moderate	47.23 (9.37)	−3.84 (−5.30, −2.38)	−3.74 (−5.24, −2.23)	−1.87 (−3.10, −0.65)
Severe	43.03 (10.93)	−7.94 (−9.50, −6.42)	−7.52 (−9.12, −5.93)	−2.59 (−3.94, −1.25)
*p*‐trend		< 0.01	< 0.01	< 0.01
Sleep efficiency				
≥ 85%	49.34 (9.82)	0 [reference]	0 [reference]	0 [reference]
75%–84%	48.02 (9.48)	−1.54 (−2.76, −0.33)	−1.24 (−2.49, 0.02)	−0.51 (−1.51, 0.49)
65%–74%	46.47 (9.65)	−3.09 (−4.54, −1.63)	−2.78 (−4.28, −1.28)	−0.31 (−1.51, 0.90)
< 65%	44.30 (10.95)	−4.95 (−6.47, −3.42)	−4.43 (−6.02, −2.83)	−0.44 (−1.74, 0.86)
*p*‐trend		< 0.01	< 0.01	0.45
Sleep disturbance				
Mild	50.00 (9.16)	0 [reference]	0 [reference]	0 [reference]
Severe	45.34 (10.39)	−4.63 (−5.61, −3.65)	−4.14 (−5.18, −3.10)	−0.77 (−1.64, 0.11)
*p*‐trend		< 0.01	< 0.01	0.74
Sleep medication				
Not during the past month	48.88 (9.80)	0 [reference]	0 [reference]	0 [reference]
Less than once a week	47.40 (9.43)	−1.58 (−3.23, 0.07)	−1.71 (−3.41, −0.00)	−1.00 (−2.35, 0.36)
Once or twice a week	46.16 (10.76)	−3.16 (−4.95, −1.38)	−3.15 (−5.00, −1.29)	−0.71 (−2.19, 0.77)
Three or more times a week	44.94 (10.16)	−4.02 (−5.40, −2.63)	−3.66 (−5.09, −2.23)	−0.80 (−1.95, 0.36)
*p*‐trend		< 0.01	< 0.01	< 0.01
Day time dysfunction				
None	54.45 (6.70)	0 [reference]	0 [reference]	0 [reference]
Mild	46.40 (9.13)	−7.84 (−8.82, −6.85)	−7.96 (−8.98, −6.94)	−4.90 (−5.83, −3.97)
Moderate	38.15 (10.55)	−15.55 (−17.19, −13.90)	−15.41 (−17.15, −13.67)	−6.89 (−8.57, −5.21)
Severe	35.26 (10.40)	−18.68 (−22.24, −15.11)	−18.42 (−22.25, −14.59)	−8.67 (−12.08, −5.25)
*p*‐trend		< 0.01	< 0.01	< 0.01

Abbreviations: CI, confidence intervals; MV, multivariable model.

^a^ Adjusted for age (years).

^b^ Multivariable (MV) model additionally adjusted study location (Edmonton, Calgary), marital status (married or common‐law, widowed/separated/divorced, single/never married), ethnicity (White, non‐white), education attainment (high school or below, college, university, graduate school), annual family income (< $50,000, $50,000–$100,000, $100,000–$150,000, $150,000), lean/fat mass ratio (kg/m^2^), total caloric intake (kcal/day), moderate to vigorous intensity physical activity (min/day), alcohol consumed (g/day), smoking (never smoker, past smoker, current smoker), disease stage (I, II, III), tumor grade (1, 2, 3), surgery status (pre‐surgery [neoadjuvant therapy], lumpectomy, mastectomy), and comorbidity score (0–8) obtained from the Charlson Comorbidity Index.

^c^ Additionally adjusted for depressive symptoms (0–21) obtained from the Patient Health Questionnaire (PHQ‐9).

^d^ Good and poor sleepers were defined by global PSQI scores (obtained through the Pittsburgh Quality Index questionnaire), with values of 8 or less corresponding to good sleepers, and values > 8 corresponding to poor sleepers.

The associations between all measures of sleep and QoL were nearly all greater for MCS than PCS. Poor global sleep quality was associated with statistically significant and clinically meaningful lower physical (*β* = −3.0, 95% CI: −3.8 to −2.3) and mental (*β* = −5.7, 95% CI: −6.7 to −4.7) well‐being. Except for sleep onset timing, all PSQI subscales were statistically significantly associated with PCS, whilst only poor sleep quality and severe daytime dysfunction exhibited clinically meaningful lower PCS. Similar to PCS, all PSQI subscales, except for sleep onset timing, were associated with MCS, and these associations were statistically significant and clinically meaningful.

### 3.3 Depressive Symptoms

After adjusting for depressive symptoms as a covariate in the multivariable regression models, the associations of the global sleep quality and its PSQI subscale scores with QoL were weakened for both PCS and MSC in terms of both effect sizes and *p*‐trend (Tables 2 and 3). The PSQI subscale that remained associated with clinically meaningfully lower PCS was daytime dysfunction at the moderate (*β* = −4.1, 95% CI = −5.7 to −2.6) or severe (*β* = −8.3, 95% CI = −11.4 to −5.1) levels. Likewise, daytime dysfunction remained associated with lower MCS after adjusting for depressive symptoms, and these associations were clinically meaningful at all levels of dysfunction (mild: *β* = −4.9, 95% CI = −5.7 to −4.0; moderate: *β* = −6.9, 95% CI = −8.6 to −5.2; severe: *β* = −8.7, 95% CI = −12.1 to −5.3).

To assess potential effect modification, we tested an interaction term between depressive symptoms and global sleep quality. Given a significant interaction between global sleep quality and depressive symptoms in relation to MCS, but not PCS, we further conducted stratified analyses by depressive symptoms for MCS (Table S3). Similar to the main analyses, daytime dysfunction remained associated with lower MCS. These associations were clinically meaningful from mild to severe daytime dysfunction and appeared to be stronger for women with any depressive symptoms compared with those who had none to minimal depressive symptoms. In addition, there was indication that moderate (31–60 min, *β* = −3.3, 95% CI = −4.6 to −1.9) or severe (> 60 min, *β* = −3.2, 95% CI = −4.9 to −1.5) sleep latency was associated with clinically meaningfully lower MSC among women with none to minimal depressive symptoms.

### 3.4 Sensitivity Analyses

Estimates from sensitivity analyses in participant subgroups defined by treatment status (active vs. no treatment) were generally similar (Tables S4 and S5). After adjusting for depressive symptoms, only daytime dysfunction appeared to be consistently associated with clinically meaningful lower PCS and MCS. In addition, there were no noticeable differences in these associations by treatment status.

## 4 Discussion

In this large sample of women newly diagnosed with breast cancer, approximately 43% reported poor global sleep quality (i.e., were classified as poor sleepers) and 10.5% had clinically meaningful depressive symptoms. Poor global sleep quality and most PSQI subscales were associated with clinically meaningful impairments in both physical and mental well‐being aspects of QoL. However, these associations were substantially attenuated after adjusting for depressive symptoms and in subgroup analyses stratified by the presence or absence of depressive symptoms. Notably, daytime dysfunction remained significantly associated with worse physical and mental well‐being, even after accounting for depressive symptoms.

Our findings of the high prevalence of sleep problems and its association with worse QoL are consistent with previous studies conducted among breast cancer survivors [7, 12, 21], particularly a stronger association observed with mental (MCS) compared to physical (PCS) well‐being [8]. While prior studies have reported associations between sleep and depression [35] and between sleep and QoL [9, 10, 37], respectively, most have not examined the role of depressive symptoms in the relationship between sleep and QoL [6, 7, 8, 9, 10, 11, 12]. Studies previously noted that persistent insomnia may contribute to the development of depression, and in turn, impair QoL [7]. Despite recognizing a possible bi‐directional relationship between sleep and depression in both the general population and among breast cancer survivors [13, 14, 15], to our knowledge, no study has directly accounted for depressive symptoms when analyzing associations between sleep and QoL.

We extend previous work by explicitly examining the role of depressive symptoms in the relationship between sleep and QoL as understanding this interplay is critical for guiding intervention strategies. If depressive symptoms act as a confounder, both sleep and depression may need to be addressed as separate yet co‐occurring targets in the intervention design. If depressive symptoms function as an effect modifier, sleep interventions may need to be tailored according to the severity of depressive symptoms. Alternatively, if depressive symptoms act as a mediator, improving sleep could help prevent or reduce depressive symptoms, thereby enhancing overall QoL. Clarifying these pathways is essential for optimizing intervention approaches to address the complex interrelations among sleep, depression, and QoL in breast cancer survivorship.

In the present analyses, we found that adjusting for depressive symptoms substantially attenuated the associations between poor sleep and QoL, suggesting that depressive symptoms play an important role in this relationship. Clinically, this finding implies that improving QoL may require addressing both sleep disturbances and depression as co‐occurring but distinct targets (i.e., confounders). Notably, the prevalence of poor global sleep quality (43%) was over four times higher than that of clinically meaningful depressive symptoms (10.5%). This difference aligns with previous research suggesting that sleep problems may emerge earlier than depression or arise independently but subsequently contribute to its development through biologic pathways such as elevated inflammation responses [38, 39, 40, 41]. As such, sleep problems could serve as an early sign of mental health deterioration. However, given the cross‐sectional nature of our analyses, we are unable to determine the temporal relationship between sleep problems and depressive symptoms. It remains possible that depressive symptoms may mediate the relationship between sleep and QoL. Longitudinal studies are needed to clarify these pathways and inform the design of more precisely targeted interventions.

In our analyses, the impaired physical and mental well‐being associated with poor global sleep quality and several other PSQI subscales were largely attenuated after adjusting for or stratified by depressive symptoms. In contrast, the PSQI subscale daytime dysfunction remained significantly associated with clinically meaningful impairments in both physical and mental aspects of QoL. This finding suggests that poor sleep‐induced functional limitation may exert an independent, yet clinically significant burden on QoL, beyond mood symptoms. For women newly diagnosed with breast cancer, targeting daytime dysfunction via improvements in sleep hygiene may represent a critical opportunity for improving QoL.

A major strength of this study is the large sample size of newly diagnosed breast cancer patients with data collected using instruments that have been validated in cancer populations, allowing for robust statistical analyses. Additionally, we had high‐quality data on potential confounding factors such as body composition, device‐based measures of physical activity via accelerometers, and cancer data obtained through medical chart abstractions, which adds rigor to the study findings. We provided estimates on the clinically meaningful difference in PCS and MCS scores specific to each aspect of sleep health, highlighting the importance of addressing poor sleep‐induced daytime dysfunction during breast cancer survivorship. The causal inference on our present findings is limited by the cross‐sectional design, but our evaluation of the potential role of depressive symptoms in the relationship between sleep and QoL underscores a need for mediation analyses using longitudinal data to inform targeted interventions. Another limitation lies in the nonuniform timing of the assessments. Our participants were recruited and assessed within 90 days of surgery and were asked to recall their experiences over the past 14–30 days. Therefore, responses to these assessments may reflect experiences ranging from shortly before pre‐diagnosis (i.e., study enrollment within 30 days of diagnosis), post‐diagnosis, and during adjuvant therapy. Nevertheless, a stratified analysis of active treatment and no treatment groups showed mostly no significant differences.

## 5 Conclusion

In this large sample of women with newly diagnosed breast cancer, nearly half experienced poor sleep quality and 10% were affected by clinically meaningful depression. Poor sleep quality was associated with clinically meaningful impairments in both physical and mental well‐being aspects of QoL. However, these associations were substantially attenuated after accounting for depressive symptoms. Longitudinal analyses are needed to examine whether depressive symptoms mediate the relationship between sleep and QoL, to inform the design of interventions to improve the QoL during breast cancer survivorship.

## Author Contributions

**Lin Yang:** conceptualization (lead), data curation (supporting), formal analysis (supporting), funding acquisition (supporting), investigation (lead), methodology (lead), project administration (equal), supervision (lead), writing – original draft (lead), writing – review and editing (equal). **Temoor Tayyab:** data curation (equal), formal analysis (lead), investigation (supporting), methodology (supporting), writing – original draft (equal), writing – review and editing (equal). **Renée L. Kokts‐Porietis:** data curation (equal), formal analysis (supporting), investigation (supporting), methodology (equal), writing – original draft (equal), writing – review and editing (equal). **Qinggang Wang:** data curation (equal), formal analysis (supporting), methodology (supporting), writing – review and editing (equal). **Jessica McNeil:** data curation (supporting), investigation (equal), methodology (equal), writing – review and editing (equal). **Charles E. Matthews:** conceptualization (supporting), data curation (supporting), formal analysis (supporting), investigation (equal), methodology (equal), writing – review and editing (equal). **Leanne Dickau:** data curation (equal), methodology (supporting), writing – review and editing (equal). **Jeff K. Vallance:** conceptualization (supporting), data curation (supporting), formal analysis (supporting), funding acquisition (supporting), investigation (equal), methodology (equal), writing – review and editing (equal). **Margaret L. McNeely:** conceptualization (supporting), data curation (supporting), funding acquisition (supporting), investigation (equal), methodology (equal), writing – review and editing (equal). **S. Nicole Culos‐Reed:** conceptualization (supporting), data curation (supporting), funding acquisition (supporting), investigation (equal), methodology (equal), writing – review and editing (equal). **Karen A. Kopciuk:** conceptualization (supporting), formal analysis (supporting), funding acquisition (supporting), investigation (equal), methodology (equal), writing – review and editing (equal). **Kerry S. Courneya:** conceptualization (supporting), data curation (lead), funding acquisition (lead), investigation (equal), methodology (equal), supervision (equal). **Christine M. Friedenreich:** conceptualization (supporting), data curation (lead), funding acquisition (lead), investigation (equal), methodology (equal), project administration (equal), resources (equal), supervision (equal), writing – review and editing (equal).

## Disclosure

The funders had no role in the design and conduct of the study; collection, management, analysis, and interpretation of the data; and preparation, review, or approval of the manuscript.

## Conflicts of Interest

The authors declare no conflicts of interest.

## Supporting information


**Table S1:** Adjuvant therapy and reproductive characteristics of the AMBER cohort at baseline (*N* = 1454).
**Table S2:**: Pittsburgh Sleep Quality Index (PSQI) subscale scores in the AMBER cohort at baseline (*N* = 1454).
**Table S3:**. Multivariable‐adjusted associations of sleep characteristics with SF‐36 measured quality of life, Mental Component Score (MCS) stratified by depression severity.
**Table S4:** Multivariable‐adjusted associations of sleep characteristics with SF‐36 measured quality of life, Physical Component Score (PCS) stratified by treatment status.
**Table S5:** Multivariable‐adjusted associations of sleep characteristics with SF‐36 measured quality of life, Mental Component Score (MCS) stratified by treatment status.

## Data Availability

The data that support the findings on this study are available from the corresponding author upon reasonable request.
