# Supplementary material for: Sleep, Depressive Symptoms, and Quality of Life Among Women With Newly Diagnosed Breast Cancer: Baseline Results From the AMBER Cohort Study
Source: Cancer Med. 2026 Feb 12;15(2):e71586. doi: 10.1002/cam4.71586 (PMC12900079; doi:10.1002/cam4.71586)
Supplement: Supplementary file 1 — Table S1: Adjuvant therapy and reproductive characteristics of the AMBER cohort at baseline (N = 1454). Table S2:: Pittsburgh Sleep Quality Index (PSQI) subscale scores in the AMBER cohort at baseline (N = 1454). Table S3:. Multivariable‐adjusted associations of sleep characteristics with SF‐36 measured quality of life, Mental Component Score (MCS) stratified by depression severity. Table S4: Multivariable‐adjusted associations of sleep characteristics with SF‐36 measured quality of life, Physical Component Score (PCS) stratified by treatment status. Table S5: Multivariable‐adjusted associations of sleep characteristics with SF‐36 measured quality of life, Mental Component Score (MCS) stratified by treatment status. [file CAM4-15-e71586-s001.docx]

| **Supplementary Table 1: Adjuvant therapy and reproductive characteristics of the AMBER cohort at baseline (N=1,454)**^a^ | | | |
| --- | --- | --- | --- |
|  | **Overall (N=1454)** | **Good sleeper (N=828)** | **Poor sleeper (N=626)** |
| **Radiation** |  |  |  |
| After baseline | 946 (65.1%) | 553 (66.8%) | 393 (62.8%) |
| Before baseline | 131 (9.0%) | 75 (9.1%) | 56 (8.9%) |
| Missing | 1 (0.1%) | 0 (0%) | 1 (0.2%) |
| No radiation | 376 (25.9%) | 200 (24.2%) | 176 (28.1%) |
| **Chemotherapy** |  |  |  |
| After baseline | 371 (25.5%) | 205 (24.8%) | 166 (26.5%) |
| Before baseline | 472 (32.5%) | 262 (31.6%) | 210 (33.5%) |
| Missing | 1 (0.1%) | 0 (0%) | 1 (0.2%) |
| No chemotherapy | 610 (42.0%) | 361 (43.6%) | 249 (39.8%) |
| **Hysterectomy** |  |  |  |
| No | 1187 (81.6%) | 700 (84.5%) | 487 (77.8%) |
| Yes | 267 (18.4%) | 128 (15.5%) | 139 (22.2%) |
| **Ovaries removed** |  |  |  |
| No | 1360 (93.5%) | 785 (94.8%) | 575 (91.9%) |
| Yes | 94 (6.5%) | 43 (5.2%) | 51 (8.1%) |
| **Menopausal status** |  |  |  |
| Post-menopausal | 858 (59.0%) | 479 (57.9%) | 379 (60.5%) |
| Pre-menopausal | 596 (41.0%) | 349 (42.1%) | 247 (39.5%) |
| **Birth Control** |  |  |  |
| No | 1315 (90.4%) | 742 (89.6%) | 573 (91.5%) |
| Yes | 139 (9.6%) | 86 (10.4%) | 53 (8.5%) |
| **Parity** |  |  |  |
| 0 | 293 (20.2%) | 160 (19.3%) | 133 (21.2%) |
| 1 | 171 (11.8%) | 97 (11.7%) | 74 (11.8%) |
| ≥ 2 | 990 (68.1%) | 571 (69.0%) | 419 (66.9%) |
| **Hormone therapy** |  |  |  |
| No | 1269 (87.3%) | 751 (90.7%) | 518 (82.7%) |
| Yes | 185 (12.7%) | 77 (9.3%) | 108 (17.3%) |
| ^a^ Good and poor sleepers were defined by global PSQI scores (obtained through the Pittsburgh Quality Index questionnaire), with values of 8 or less corresponding to good sleepers, and values greater than 8 corresponding to poor sleepers | | | |

| **Supplementary Table 2:** **Pittsburgh Sleep Quality Index (PSQI) subscale scores in the AMBER cohort at baseline (N=1,454)**^a^ | | | |
| --- | --- | --- | --- |
|  | **Overall (N=1454)** | **Good sleeper (N=828)** | **Poor sleeper (N=626)** |
| **PSQI 1: Sleep quality** |  |  |  |
| Very good | 232 (16.0%) | 227 (27.4%) | 5 (0.8%) |
| Fairly good | 791 (54.4%) | 554 (66.9%) | 237 (37.9%) |
| Fairly bad | 359 (24.7%) | 46 (5.6%) | 313 (50.0%) |
| Very bad | 72 (5.0%) | 1 (0.1%) | 71 (11.3%) |
| **PSQI 2: Sleep latency** |  |  |  |
| None | 333 (22.9%) | 296 (35.7%) | 37 (5.9%) |
| Mild | 554 (38.1%) | 402 (48.6%) | 152 (24.3%) |
| Moderate | 305 (21.0%) | 118 (14.3%) | 187 (29.9%) |
| Severe | 262 (18.0%) | 12 (1.4%) | 250 (39.9%) |
| **PSQI 3: Sleep duration** |  |  |  |
| ≤ 6 h/d | 440 (30.3%) | 90 (10.9%) | 350 (55.9%) |
| ≥ 9 h/d | 161 (11.1%) | 135 (16.3%) | 26 (4.2%) |
| 6-9 h/d | 853 (58.7%) | 603 (72.8%) | 250 (39.9%) |
| **PSQI 4: Sleep efficiency** |  |  |  |
| ≥ 85% | 646 (44.4%) | 561 (67.8%) | 85 (13.6%) |
| 75-84% | 382 (26.3%) | 217 (26.2%) | 165 (26.4%) |
| 65-74% | 226 (15.5%) | 45 (5.4%) | 181 (28.9%) |
| <65% | 200 (13.8%) | 5 (0.6%) | 195 (31.2%) |
| **PSQI 5: Sleep disturbances** |  |  |  |
| 0-1 | 784 (53.9%) | 596 (72.0%) | 188 (30.0%) |
| 2-3 | 670 (46.1%) | 232 (28.0%) | 438 (70.0%) |
| **PSQI 6: Sleep medication** |  |  |  |
| Not during the past month | 941 (64.7%) | 681 (82.2%) | 260 (41.5%) |
| Less than once a week | 152 (10.5%) | 83 (10.0%) | 69 (11.0%) |
| Once or twice a week | 127 (8.7%) | 30 (3.6%) | 97 (15.5%) |
| Three or more times a week | 234 (16.1%) | 34 (4.1%) | 200 (31.9%) |
| **PSQI 7: Daytime dysfunction** |  |  |  |
| None | 434 (29.8%) | 356 (43.0%) | 78 (12.5%) |
| Mild | 861 (59.2%) | 436 (52.7%) | 425 (67.9%) |
| Moderate | 136 (9.4%) | 35 (4.2%) | 101 (16.1%) |
| Severe | 23 (1.6%) | 1 (0.1%) | 22 (3.5%) |
| ^a^ Good and poor sleepers were defined by global PSQI scores (obtained through the Pittsburgh Quality Index questionnaire), with values of 8 or less corresponding to good sleepers, and values greater than 8 corresponding to poor sleepers | | | |

| **Supplementary Table 3. Multivariable-Adjusted Associations of Sleep Characteristics with SF-36 Measured Quality of Life, Mental Component Score (MCS) Stratified by Depression Severity.** | | | | | | | | |
| --- | --- | --- | --- | --- | --- | --- | --- | --- |
|  | | **None-Minimal (n=913)** | | |  | **Severe (n=541)** | | |
|  | | **Mean MCS (SD)** |  | **MV model^a^** |  | **Mean MCS (SD)** |  | **MV model^a^** |
| **Sleep profile** ^b^ | |  |  |  |  |  |  |  |
| Good sleepers | | 53.09 (6.60) |  | 0 [reference] |  | 40.85 (10.07) |  | 0 [reference] |
| Poor sleepers | | 50.75 (7.38) |  | -2.19 (-3.21, -1.18) |  | 39.80 (9.52) |  | -0.87 (-2.71, 0.97) |
| p-trend | |  |  | <0.01 |  |  |  | 0.04 |
| **Sleep quality** | |  |  |  |  |  |  |  |
| Very good | | 54.47 (6.75) |  | 0 [reference] |  | 42.25 (10.51) |  | 0 [reference] |
| Fairly good | | 52.29 (6.46) |  | -1.90 (-3.03, -0.76) |  | 40.72 (9.59) |  | -0.84 (-4.42, 2.74) |
| Fairly bad | | 49.98 (8.15) |  | -3.74 (-5.29, -2.19) |  | 39.87 (9.51) |  | -1.49 (-5.08, 2.11) |
| Very bad | | 52.65 (5.81) |  | -1.67 (-5.68, 2.34) |  | 37.76 (10.10) |  | -4.25 (-8.45, -0.05) |
| p-trend | |  |  | <0.01 |  |  |  | 0.02 |
| **Sleep duration** | |  |  |  |  |  |  |  |
| 6-9 h/d | | 52.68 (6.81) |  | 0 [reference] |  | 40.88 (9.19) |  | 0 [reference] |
| ≤ 6 h/d | | 51.76 (7.11) |  | -0.89 (-2.02, 0.25) |  | 39.78 (10.11) |  | -1.09 (-2.91, 0.72) |
| ≥ 9 h/d | | 52.26 (7.07) |  | -0.48 (-1.98, 1.03) |  | 38.56 (9.95) |  | -2.51 (-5.29, 0.28) |
| **Sleep timing** | |  |  |  |  |  |  |  |
| < 10 pm | | 52.13 (7.10) |  | 0 [reference] |  | 38.75 (9.10) |  | 0 [reference] |
| 10-11 pm | | 52.23 (7.12) |  | -0.03 (-1.26, 1.20) |  | 40.23 (9.76) |  | 0.98 (-1.34, 3.31) |
| 11-12 pm | | 53.04 (6.22) |  | 0.71 (-0.60, 2.02) |  | 40.81 (9.31) |  | 1.65 (-0.79, 4.09) |
| ≥ 12 pm | | 51.87 (7.72) |  | -0.24 (-2.13, 1.66) |  | 40.38 (11.39) |  | 1.98 (-1.26, 5.23) |
| p-trend | |  |  | 0.44 |  |  |  | 0.60 |
| **Sleep latency** | |  |  |  |  |  |  |  |
| None | | 54.04 (6.02) |  | 0 [reference] |  | 39.37 (10.05) |  | 0 [reference] |
| Mild | | 52.31 (6.57) |  | -1.95 (-3.05, -0.85) |  | 40.72 (9.91) |  | 1.23 (-1.59, 4.06) |
| Moderate | | 51.19 (7.59) |  | -3.25 (-4.55, -1.94) |  | 41.04 (8.52) |  | 2.29 (-0.72, 5.30) |
| Severe | | 50.65 (8.24) |  | -3.19 (-4.90, -1.48) |  | 39.18 (10.08) |  | -0.01 (-2.87, 2.85) |
| p-trend | |  |  | <0.01 |  |  |  | 0.70 |
| **Sleep efficiency** | |  |  |  |  |  |  |  |
| ≥ 85% | | 53.00 (6.93) |  | 0 [reference] |  | 39.65 (9.79) |  | 0 [reference] |
| 75-84% | | 51.61 (6.83) |  | -1.42 (-2.51, -0.33) |  | 41.16 (10.07) |  | 1.61 (-0.67, 3.90) |
| 65-74% | | 52.05 (7.02) |  | -0.91 (-2.37, 0.54) |  | 40.89 (8.65) |  | 0.94 (-1.43, 3.31) |
| <65% | | 52.20 (6.66) |  | -0.66 (-2.37, 1.03) |  | 39.03 (10.06) |  | -0.61 (-2.96, 1.74) |
| p-trend | |  |  | 0.13 |  |  |  | 0.69 |
| **Sleep disturbance** | |  |  |  |  |  |  |  |
| Mild | | 52.85 (6.96) |  | 0 [reference] |  | 40.98 (9.45) |  | 0 [reference] |
| Severe | | 51.65 (6.74) |  | -1.27 (-2.26, -0.29) |  | 39.68 (9.82) |  | -0.97 (-2.82, 0.87) |
| p-trend | |  |  | <0.01 |  |  |  | 0.15 |
| **Sleep medication** | |  |  |  |  |  |  |  |
| Not during the past month | | 52.75 (6.90) |  | 0 [reference] |  | 40.27 (9.82) |  | 0 [reference] |
| Less than once a week | | 51.81 (6.79) |  | -1.49 (-3.00, 0.02) |  | 39.61 (8.35) |  | -1.18 (-4.04, 1.68) |
| Once or twice a week | | 52.48 (6.72) |  | -0.85 (-2.68, 0.98) |  | 39.93 (10.38) |  | -0.69 (-3.44, 2.06) |
| Three or more times a week | | 50.95 (7.03) |  | -2.23 (-3.69, -0.77) |  | 40.14 (9.73) |  | -0.06 (-2.15, 2.03) |
| p-trend | |  |  | <0.01 |  |  |  | 0.87 |
| **Day time dysfunction** | |  |  |  |  |  |  |  |
| None | | 55.13 (5.97) |  | 0 [reference] |  | 46.25 (9.33) |  | 0 [reference] |
| Mild | | 50.44 (6.75) |  | -5.08 (-5.95, -4.22) |  | 41.03 (9.11) |  | -4.78 (-8.19, -1.37) |
| Moderate | | 47.60 (9.63) |  | -8.02 (-11.13, -4.90) |  | 36.80 (10.00) |  | -8.59 (-12.30, -4.88) |
| Severe | | 46.09 (1.82) |  | -8.98 (-16.21, -1.75) |  | 32.98 (10.00) |  | -12.33 (-17.91, -6.74) |
| p-trend | |  |  | <0.01 |  |  |  | <0.01 |
|  | *Note.* CI = Confidence Intervals; MV = Multivariable model ^a^ Multivariable (MV) model adjusted for age, study location (Edmonton, Calgary), marital status (married or common-law, windowed/separated/divorced, single/never married), ethnicity (White, non-white), education attainment (high school or below, college, university, graduate school), annual family income (<$50,000, $50,000-$100,000, $100,000-$150,000 $150,000), lean/fat mass ratio (kg/m^2^), total caloric intake (kcal/day), moderate to vigorous intensity physical activity (minutes/day), alcohol consumed (g/day), smoking (never smoker, past smoker, current smoker), disease stage (I, II, III), tumor grade (1, 2, 3), surgery status (pre-surgery [neoadjuvant therapy], lumpectomy, mastectomy), and comorbidity score (0-8) obtained from the Charlson Comorbidity Index. ^b^ Good and poor sleepers were defined by global PSQI scores (obtained through the Pittsburgh Quality Index questionnaire), with values of 8 or less corresponding to good sleepers, and values greater than 8 corresponding to poor sleepers. | | | | | | | |

| **Supplementary Table 4. Multivariable-Adjusted Associations of Sleep Characteristics with SF-36 Measured Quality of Life, Physical Component Score (PCS) Stratified by Treatment Status.** | | | | | | | | |
| --- | --- | --- | --- | --- | --- | --- | --- | --- |
|  | | **Active treatment (n=597)** | | |  | **No treatment (n=856)** | | |
|  | | **Mean PCS (SD)** |  | **MV model^a^** |  | **Mean PCS (SD)** |  | **MV model^a^** |
| **Sleep profile** ^b^ | |  |  |  |  |  |  |  |
| Good sleepers | | 50.32 (7.13) |  | 0 [reference] |  | 51.32 (7.07) |  | 0 [reference] |
| Poor sleepers | | 46.75 (7.18) |  | -1.79 (-3.03, 0.55) |  | 47.18 (7.66) |  | -2.46 (-3.52, -1.40) |
| p-trend | |  |  | 0.05 |  |  |  | <0.01 |
| **Sleep quality** | |  |  |  |  |  |  |  |
| Very good | | 49.65 (8.45) |  | 0 [reference] |  | 51.93 (6.91) |  | 0 [reference] |
| Fairly good | | 49.61 (6.74) |  | -0.27 (-1.90, 1.36) |  | 50.09 (7.40) |  | -1.01 (-2.38, 0.36) |
| Fairly bad | | 46.96 (7.59) |  | -0.37 (-2.33, 1.57) |  | 47.62 (7.75) |  | -2.43 (-4.05, -0.80) |
| Very bad | | 44.57 (6.73) |  | -1.85 (-5.02, 1.32) |  | 46.24 (8.04) |  | -3.50 (-6.05, -0.96) |
| p-trend | |  |  | 0.39 |  |  |  | <0.01 |
| **Sleep duration** | |  |  |  |  |  |  |  |
| 6-9 h/d | | 49.26 (6.84) |  | 0 [reference] |  | 50.49 (7.39) |  | 0 [reference] |
| ≤ 6 h/d | | 48.06 (7.88) |  | 0.46 (-0.85, 1.77) |  | 47.93 (7.56) |  | -1.25 (-2.37, -0.12) |
| ≥ 9 h/d | | 47.94 (8.36) |  | -0.89 (-2.72, 0.95) |  | 49.10 (8.12) |  | -0.64 (-2.20, 0.93) |
| **Sleep timing** | |  |  |  |  |  |  |  |
| < 10 pm | | 49.00 (7.05) |  | 0 [reference] |  | 49.79 (7.70) |  | 0 [reference] |
| 10-11 pm | | 49.16 (7.24) |  | -0.17 (-1.65, 1.32) |  | 49.76 (7.40) |  | -0.62 (-1.96, 0.71) |
| 11-12 pm | | 48.31 (7.42) |  | -0.26 (-1.85, 1.33) |  | 49.86 (7.60) |  | 0.28 (-1.10, 1.67) |
| ≥ 12 pm | | 47.61 (8.51) |  | 0.03 (-2.40, 2.45) |  | 47.63 (8.03) |  | -0.98 (-2.84, 0.88) |
| p-trend | |  |  | 0.86 |  |  |  | 0.29 |
| **Sleep latency** | |  |  |  |  |  |  |  |
| None | | 49.90 (7.46) |  | 0 [reference] |  | 50.83 (6.66) |  | 0 [reference] |
| Mild | | 49.08 (7.48) |  | -0.07 (-1.59, 1.46) |  | 50.06 (7.63) |  | -0.19 (-1.45, 1.06) |
| Moderate | | 48.77 (7.04) |  | -0.29 (-1.95, 1.37) |  | 49.01 (7.94) |  | -0.99 (-2.49, 0.50) |
| Severe | | 46.58 (7.04) |  | -0.41 (-2.31, 1.48) |  | 47.50 (7.88) |  | -0.92 (-2.52, 0.68) |
| p-trend | |  |  | 0.62 |  |  |  | 0.14 |
| **Sleep efficiency** | |  |  |  |  |  |  |  |
| ≥ 85% | | 49.64 (7.42) |  | 0 [reference] |  | 50.88 (7.26) |  | 0 [reference] |
| 75-84% | | 48.98 (7.13) |  | -0.12 (-1.51, 1.27) |  | 49.33 (7.89) |  | -0.95 (-2.12, 0.22) |
| 65-74% | | 48.09 (7.55) |  | 0.22 (-1.40, 1.86) |  | 47.96 (7.52) |  | -1.82 (-3.28, -0.36) |
| <65% | | 46.35 (6.89) |  | -1.07 (-2.86, 0.72) |  | 47.26 (7.44) |  | -1.74 (-3.28, -0.19) |
| p-trend | |  |  | 0.42 |  |  |  | <0.01 |
| **Sleep disturbance** | |  |  |  |  |  |  |  |
| Mild | | 50.50 (6.74) |  | 0 [reference] |  | 51.14 (7.21) |  | 0 [reference] |
| Severe | | 46.73 (7.53) |  | -2.31 (-3.48, -1.15) |  | 47.72 (7.65) |  | -1.73 (-2.78, -0.69) |
| p-trend | |  |  | <0.01 |  |  |  | <0.01 |
| **Sleep medication** | |  |  |  |  |  |  |  |
| Not during the past month | | 49.15 (7.35) |  | 0 [reference] |  | 50.55 (7.51) |  | 0 [reference] |
| Less than once a week | | 49.56 (6.42) |  | 1.03 (-0.83, 2.89) |  | 48.50 (6.97) |  | -1.37 (-2.97, 0.23) |
| Once or twice a week | | 46.79 (7.53) |  | -1.37 (-3.53, 0.78) |  | 48.98 (7.47) |  | -0.03 (-1.74, 1.66) |
| Three or more times a week | | 47.53 (7.70) |  | -0.42 (-2.01, 1.16) |  | 46.68 (7.65) |  | -2.04 (-3.40, -0.68) |
| p-trend | |  |  | 0.44 |  |  |  | <0.01 |
| **Day time dysfunction** | |  |  |  |  |  |  |  |
| None | | 52.16 (6.37) |  | 0 [reference] |  | 52.20 (6.54) |  | 0 [reference] |
| Mild | | 48.17 (7.19) |  | -2.54 (-3.90, -1.19) |  | 48.84 (7.70) |  | -2.19 (-3.30, -1.08) |
| Moderate | | 44.01 (6.86) |  | -4.52 (-6.84, -2.21) |  | 46.56 (7.46) |  | -3.29 (-5.40, -1.19) |
| Severe | | 41.77 (7.50) |  | -9.40 (-15.76, -3.04) |  | 41.29 (6.37) |  | -8.07 (-11.74, -4.39) |
| p-trend | |  |  | <0.01 |  |  |  | <0.01 |
|  | *Note.* CI = Confidence Intervals; MV = Multivariable model ^a^ Multivariable (MV) model adjusted for age, study location (Edmonton, Calgary), marital status (married or common-law, windowed/separated/divorced, single/never married), ethnicity (White, non-white), education attainment (high school or below, college, university, graduate school), annual family income (<$50,000, $50,000-$100,000, $100,000-$150,000 $150,000), lean/fat mass ratio (kg/m^2^), total caloric intake (kcal/day), moderate to vigorous intensity physical activity (minutes/day), alcohol consumed (g/day), smoking (never smoker, past smoker, current smoker), disease stage (I, II, III), tumor grade (1, 2, 3), surgery status (pre-surgery [neoadjuvant therapy], lumpectomy, mastectomy), comorbidity score (0-8), and depressive symptoms (0-21). ^b^ Good and poor sleepers were defined by global PSQI scores (obtained through the Pittsburgh Quality Index questionnaire), with values of 8 or less corresponding to good sleepers, and values greater than 8 corresponding to poor sleepers. | | | | | | | |

| **Supplementary Table 5. Multivariable-Adjusted Associations of Sleep Characteristics with SF-36 Measured Quality of Life, Mental Component Score (MCS) Stratified by Treatment Status.** | | | | | | | | |
| --- | --- | --- | --- | --- | --- | --- | --- | --- |
|  | | **Active treatment (n=597)** | | |  | **No treatment (n=856)** | | |
|  | | **Mean MCS (SD)** |  | **MV model^a^** |  | **Mean MCS (SD)** |  | **MV model^a^** |
| **Sleep profile** ^b^ | |  |  |  |  |  |  |  |
| Good sleepers | | 50.11 (8.77) |  | 0 [reference] |  | 50.84 (9.07) |  | 0 [reference] |
| Poor sleepers | | 44.39 (9.87) |  | -0.85 (-2.26, 0.57) |  | 44.19 (10.49) |  | -1.36 (-2.55, -0.18) |
| p-trend | |  |  | 0.05 (exact) |  |  |  | <0.01 |
| **Sleep quality** | |  |  |  |  |  |  |  |
| Very good | | 51.84 (8.19) |  | 0 [reference] |  | 53.23 (8.79) |  | 0 [reference] |
| Fairly good | | 48.78 (9.07) |  | -0.87 (-2.60, 0.85) |  | 49.23 (9.11) |  | -2.32 (-3.85, -0.79) |
| Fairly bad | | 43.44 (9.95) |  | 0.14 (-1.74, 2.02) |  | 43.73 (10.48) |  | -2.80 (-4.62, -0.98) |
| Very bad | | 40.74 (9.57) |  | --1.09 (-3.24, 1.06) |  | 39.92 (11.94) |  | -2.60 (-5.44, 0.24) |
| p-trend | |  |  | 0.62 |  |  |  | 0.01 |
| **Sleep duration** | |  |  |  |  |  |  |  |
| 6-9 h/d | | 49.04 (9.08) |  | 0 [reference] |  | 49.55 (9.34) |  | 0 [reference] |
| ≤ 6 h/d | | 45.04 (9.99) |  | -0.76 (-2.24, 0.73) |  | 45.36 (11.17) |  | -0.06 (-1.32, 1.20) |
| ≥ 9 h/d | | 47.11 (10.52) |  | -1.86 (-3.94, 0.21) |  | 47.04 (10.72) |  | -0.35 (-2.09, 1.40) |
| **Sleep timing** | |  |  |  |  |  |  |  |
| < 10 pm | | 46.05 (9.97) |  | 0 [reference] |  | 48.21 (10.27) |  | 0 [reference] |
| 10-11 pm | | 47.80 (9.34) |  | 1.21 (-0.46, 2.87) |  | 48.18 (10.37) |  | -0.94 (-2.44, 0.55) |
| 11-12 pm | | 48.86 (9.42) |  | 2.78 (0.99, 4.57) |  | 48.17 (9.69) |  | -0.50 (-2.05, 1.04) |
| ≥ 12 pm | | 46.36 (10.95) |  | 2.40 (-0.32, 5.12) |  | 46.69 (11.31) |  | -1.41 (-3.49, 0.66) |
| p-trend | |  |  | 0.82 |  |  |  | 0.36 |
| **Sleep latency** | |  |  |  |  |  |  |  |
| None | | 50.30 (9.08) |  | 0 [reference] |  | 51.81 (9.05) |  | 0 [reference] |
| Mild | | 47.87 (9.18) |  | -0.87 (-2.60, 0.85) |  | 48.86 (9.75) |  | -1.86 (-3.24, -0.47) |
| Moderate | | 48.17 (9.26) |  | 0.14 (-1.74, 2.02) |  | 46.42 (9.42) |  | -3.31 (-4.96, -1.66) |
| Severe | | 42.88 (10.35) |  | -1.09 (-3.24, 1.06) |  | 43.14 (11.36) |  | -3.49 (-5.26, -1.73) |
| p-trend | |  |  | 0.62 |  |  |  | <0.01 |
| **Sleep efficiency** | |  |  |  |  |  |  |  |
| ≥ 85% | | 48.95 (9.23) |  | 0 [reference] |  | 49.58 (10.18) |  | 0 [reference] |
| 75-84% | | 47.83 (9.91) |  | 0.47 (-1.11, 2.05) |  | 48.17 (9.18) |  | -1.26 (-2.57, 0.05) |
| 65-74% | | 46.22 (9.35) |  | -0.47 (-2.32, 1.38) |  | 46.69 (9.94) |  | -0.37 (-2.00, 1.26) |
| <65% | | 44.71 (10.37) |  | 0.22 (-1.81, 2.26) |  | 43.90 (11.35) |  | -1.03 (-2.76, 0.69) |
| p-trend | |  |  | <0.01 |  |  |  | 0.24 |
| **Sleep disturbance** | |  |  |  |  |  |  |  |
| Mild | | 49.15 (9.56) |  | 0 [reference] |  | 50.57 (8.84) |  | 0 [reference] |
| Severe | | 45.80 (9.55) |  | -0.02 (-1.36, 1.32) |  | 45.02 (10.95) |  | -1.21 (-2.38, -0.04) |
| p-trend | |  |  | <0.01 |  |  |  | 0.03 |
| **Sleep medication** | |  |  |  |  |  |  |  |
| Not during the past month | | 48.32 (9.44) |  | 0 [reference] |  | 49.26 (10.04) |  | 0 [reference] |
| Less than once a week | | 47.89 (9.50) |  | -0.14 (-2.26, 1.98) |  | 47.023 (9.42) |  | -1.69 (-3.48, 0.10) |
| Once or twice a week | | 45.77 (11.28) |  | -0.82 (-3.26, 1.63) |  | 46.39 (10.49) |  | -0.58 (-2.48, 1.32) |
| Three or more times a week | | 45.34 (9.66) |  | -0.89 (-2.70, 0.92) |  | 44.67 (10.53) |  | -0.76 (-2.29, 0.76) |
| p-trend | |  |  | 0.29 |  |  |  | 0.25 |
| **Day time dysfunction** | |  |  |  |  |  |  |  |
| None | | 53.85 (6.87) |  | 0 [reference] |  | 54.81 (6.59) |  | 0 [reference] |
| Mild | | 46.63 (8.80) |  | -4.07 (-5.57, -2.56) |  | 46.20 (9.36) |  | -5.58 (-6.77, -4.39) |
| Moderate | | 38.64 (10.37) |  | -7.33 (-9.90, -4.76) |  | 37.72 (10.76) |  | -6.73 (-8.99, -4.47) |
| Severe | | 32.26 (7.26) |  | -12.83 (-19.90, -5.75) |  | 36.31 (11.30) |  | -7.20 (-11.15, -3.25) |
| p-trend | |  |  | <0.01 |  |  |  | <0.01 |
|  | *Note.* CI = Confidence Intervals; MV = Multivariable model ^a^ Multivariable (MV) model adjusted for age, study location (Edmonton, Calgary), marital status (married or common-law, windowed/separated/divorced, single/never married), ethnicity (White, non-white), education attainment (high school or below, college, university, graduate school), annual family income (<$50,000, $50,000-$100,000, $100,000-$150,000 $150,000), lean/fat mass ratio (kg/m^2^), total caloric intake (kcal/day), moderate to vigorous intensity physical activity (minutes/day), alcohol consumed (g/day), smoking (never smoker, past smoker, current smoker), disease stage (I, II, III), tumor grade (1, 2, 3), surgery status (pre-surgery [neoadjuvant therapy], lumpectomy, mastectomy), and comorbidity score (0-8), and depressive symptoms (0-21). ^b^ Good and poor sleepers were defined by global PSQI scores (obtained through the Pittsburgh Quality Index questionnaire), with values of 8 or less corresponding to good sleepers, and values greater than 8 corresponding to poor sleepers. | | | | | | | |
